# Asymptotic analysis of mathematical model describing a new treatment of breast cancer using AZD9496 and palbociclib

**DOI:** 10.3389/fonc.2024.1482223

**Published:** 2025-01-28

**Authors:** Ophir Nave

**Affiliations:** ^1^ Faculty of Computer Science, The College of Management Academic Studies, Rishon Lezion, Israel; ^2^ Department of Mathematics, Faculty of Science, Jerusalem College of Technology, Jerusalem, Israel

**Keywords:** SPVF, cancer treatment, AZD9496, palbociclib, mathematical model, asymptotic analysis method

## Abstract

**Introduction:**

Cancer is a collective name for a group of diseases consisting of dozens of different types of malignant tumors, characterized by rapid and uncontrolled proliferation of cells in the body. Cancer can start almost anywhere in the human body such as the breast, prostate, colorectal, brain, bones, lungs, bladder etc. The main differences between the different types of cancer are related to the organ in which the tumor develops and the type of cells that compose the tumor.

**Method:**

This paper focused on the breast cancer. Breast cancer is a malignant tumor that originates in the breast tissue. It is the most common malignant tumor in women. There are several types of breast cancer, but in all types early diagnosis and treatment is crucial. In this study, the treatment of breast cancer involving a combination of two drugs was investigated: the oral estrogen receptor inhibitor AZD9496 and the CDK4/6 protein inhibitor Palbociclib. The mathematical model that described the interaction between the cancer cells, the treatment, and the immune system cells includes a system of nonlinear ordinary differential equations of the firs order. In general, dynamic variables of a given system change each at a different rate. And it is not possible to know from the mathematical model which variable is fast and which is slow. Therefore, in order to reveal the hierarchy of the system of equations ,a numerical algorithm called the singularly perturbed vector field (SPVF) was applied. This algorithm transform the mathematical model to a new coordinate system in which the rate of change of each dynamic variable of the system can be known.

**Results and Discussion:**

After writing the mathematical model in new coordinates, the equilibrium point was obtained analytically. The stability of the equilibrium points is investigated, which is essential from a practical perspective. Investigating the stability of the equilibrium points allows determination of when the tumor does not continue to develop and thereby allows adjustment of treatment continuation.

## 1 Introduction

Breast cancer accounts for approximately one-third of new cancer cases among women annually. It is the most common malignant disease among women worldwide (There were 2,296,840 new cases of breast cancer in 2022 among women (1)). Currently, at least one in eight women may experience breast cancer during their lifetime. The disease occurs mainly in women but is also found in men (the risk for men is 100 times lower than that for women). In cancer, uncontrolled cell proliferation occurs in an organ of the body. Breast cancer develops in the breast tissues, usually in the ducts that carry milk to the nipple and in the glands that produce milk. The disease is caused by a set of factors, some of which are congenital or depend on age and medical history, while others are related to lifestyle. Only approximately 10% of cases are due to heredity. Examination by a doctor (sometimes, self-examination is sufficient to determine the presence of a breast lump) is essential in cases where an unfamiliar breast lump is noticed. Breast cancer awareness is key to health because the earlier the disease is diagnosed, the higher are the chances of recovery. Approximately 80% of the lumps detected in the breast are benign, i.e., not malignant, and do not pose a risk. These include fibroadenomas (lumps of fibrous tissue), cysts (fluid sacs), and congestion. However, certain benign lumps may increase the risk of developing breast cancer (2–6).

Women diagnosed with breast tumors usually undergo surgery for tumor resection (lumpectomy or partial excision of the breast) (7, 8). A sample is also obtained from the lymph nodes in the armpit (sentinel gland biopsy). Sometimes, a more extensive operation is necessary (9, 10), such as removal of the entire breast (mastectomy or complete excision) or extensive excision of lymph nodes from the armpit. In most cases, when a complete mastectomy is necessary, breast reconstruction surgery can be performed immediately (11–14).

Radiation. After surgery, complementary radiation therapy is usually required, especially if a partial excision is performed. Radiation can be administered to the entire breast, and sometimes to the lymph nodes that drain the breast. In some cases, one dose of radiation administered during the resection surgery is sufficient (15–17).

Chemotherapy. Chemotherapy includes drugs that damage tumor cells. Chemotherapy sometimes has side effects such as nausea, vomiting, and hair loss. These side effects can be alleviated by medication. Notably, every patient with breast cancer patient may not require chemotherapy. Chemotherapy can be administered before or after surgery (18–27).

Biological treatments. Unlike chemotherapy, biological treatments are more specific to tumor cells and reduce damage to the remaining body, resulting in fewer side effects. The biological drugs used against breast cancer include Herceptin and Lapatinib (28–31).

Antihormonal treatments. After completion of chemotherapy and radiation treatment, some patients are recommended complementary antihormonal treatment as pills for 5 10 years. Such treatment is suitable for women whose tumors have hormone receptors, and it aims to reduce the risk of tumor recurrence. An example of an antihormonal drug is tamoxifen (32–37).

In recent years, researchers, such as mathematicians and physicists, have also been trying to find unconventional ways of treating this disease. This usually involves the development of a mathematical model; which on one hand includes models that take as many variables and parameters related to the patient as possible and on the other hand, models that can be studied, not necessarily in a numerical manner (38–42).

The advantages of a mathematical model are that it does not require a laboratory in the initial stage but only mathematical tools. This also does not require a high budget. Another advantage is that the parameters of the system can be easily changed and adapted to different patients to allow personalized treatment. The disadvantages are that a mathematical model does not reflect reality one-to-one but only provides an approximation; however, many studies indicate that the obtained approximation is sufficiently good (43–47).

In the present study, the investigation focused on a mathematical model that describes cancer treatment using a combination of two drugs: the oral estrogen receptor inhibitor AZD9496 and a CDK4/6 protein inhibitor Palbociclib using an asymptotic method called singular perturbed vector field (SPVFM), which allows us to determine the equilibrium points of the system, which is essential from a practical viewpoint of view.

The paper is organized as follows: In the next section the mathematical model of breast cancer and its treatment is presented. Subsequently, the algorithm of singularly perturbed vector fields and its application to the mathematical model were introduced. Finally, the results of the research and their analysis are presented.

## 2 Mathematical model definition

In this section, a mathematical model derived from the article (47) is presented for ER-positive breast cancer treatment using two different drugs: AZD9496 and palbociclib. In this study, a new personalized treatment based on analytical functions dependent on two parameters is proposed: the dosage of the medicine and the time interval between treatments. These two parameters enable us to control the treatments such that the dosage and time intervals can be modified depending on the tumor size. For this purpose, an ODE equation describing the treatment function in relation to tumor size wasted. The solution profiles of these equations show the dosage and time interval as a function of the tumor size at each given time. The dynamical variables of the model are as follows: *C_C_* [*cell*] is the MCF-7 tumor cell population, *N_K_* [*cellL* ^−1^], is the NK cell population, *W_BC_* [*cellL* ^−1^] is the WBC population, *C_TL_* [*cellL* ^−1^] is the CTL population, $A_{ZD}^{nc}$ [*mg*] is the AZD9496 not in circulation, $A_{ZD}^{c}$ [*mg*] is the AZD9496 in circulation, $P_{a}^{nc}$ [*mg*] is the Palbociclib not in circulation, $P_{a}^{c}$ [*mg*] Palbociclib in circulation, ℱ [*mg*] and ℋ [*mg*] are functions of AZD9496 and Palbociclib treatments, respectively, ( $q_{A_{ZD}}$ is the amount of AZD9496, and $q_{P_{a}}$ is the amount of Palbociclib). Based on the above assumptions, the mathematical model is a system of first-order nonlinear ordinary differential equations in the form:


$$\frac{dC_{C}}{dt}=C_{C}\left(ae^{-\alpha_{9}P_{a}^{c}}+\frac{ce^{-\alpha_{10}A_{ZD}^{c}}EC_{C}}{1+\alpha_{1}E+\beta_{1}C_{C}^{2}}\right)\left(1-\frac{C_{C}}{K}\right)$$

(1) $$-\frac{p_{1}C_{C}N_{K}^{2}}{1+\alpha_{2}C_{C}+\beta_{2}N_{K}^{2}}-\frac{p_{6}C_{C}^{2}C_{TL}}{1+\alpha_{6}C_{C}^{2}+\beta_{6}C_{TL}}\equiv F_{1}\left(\vec{W}\right),$$

(2) $$\frac{dN_{K}}{dt}=eW_{BC}-fN_{K}-p_{2}N_{K}C_{C}+\frac{p_{3}N_{K}C_{C}}{1+\alpha_{3}C_{C}+\beta_{3}N_{K}}\equiv F_{2}\left(\vec{W}\right),$$

(3) $$\frac{dW_{BC}}{dt}=\alpha -\beta W_{BC}\equiv F_{3}\left(\vec{W}\right),$$

(4) $$\frac{dC_{TL}}{dt}=\left(\frac{C_{C}K_{L}-C_{TL}C_{C}}{K_{L}\left(\alpha_{5}+C_{C}\right)}\right)\left(p_{4}L_{N}+\frac{p_{5}I}{\alpha_{4}+I}C_{TL}\right)-dC_{TL}\equiv F_{4}\left(\vec{W}\right),$$

(5) $$\frac{dA_{ZD}^{nc}}{dt}=-\alpha_{7}A_{ZD}^{nc}+ℱ\left(t\right)\equiv F_{5}\left(\vec{W}\right),$$

(6) $$\frac{dA_{ZD}^{c}}{dt}=\alpha_{7}A_{ZD}^{nc}-\beta_{4}A_{ZD}^{c}\equiv F_{6}\left(\vec{W}\right),$$

(7) $$\frac{dP_{a}^{nc}}{dt}=-\alpha_{8}P_{a}^{nc}+ℋ\left(t\right)\equiv F_{7}\left(\vec{W}\right),$$

(8) $$\frac{dP_{a}^{c}}{dt}=\alpha_{8}P_{a}^{nc}-\beta_{5}P_{a}^{c}\equiv F_{8}\left(\vec{W}\right),$$

(9) $$\frac{dℱ}{dt}={ℱ}^{\epsilon_{1}}\left(t\right)C_{C}-{ℱ}^{\epsilon_{2}}\left(t\right)\equiv F_{9}\left(\vec{W}\right),$$

(10) $$\frac{dℋ}{dt}={ℋ}^{\epsilon_{3}}\left(t\right)C_{C}-{ℋ}^{\epsilon_{4}}\left(t\right)\equiv F_{10}\left(\vec{W}\right)$$

The initial conditions of the model at t=0 are:


(11) $$\begin{array}{l} C_{C}=8.72\cdot 10^{7},N_{K}=2.5\cdot 10^{8},W_{BC}=4.3\cdot 10^{9},C_{TL}=6.6\cdot 10^{8},A_{ZD}^{nc}=0,\\ A_{ZD}^{c}=0,P_{a}^{nc}=0,P_{a}^{c}=0,ℱ\left(0,q_{A_{ZD}}\right)=q_{0}^{1},ℋ\left(0,q_{P_{a}}\right)=q_{0}^{2}. \end{array}$$

The vector $\vec{W}$ will be define in section 4.1. The following parameters are used for numerical simulations of the application.

***Parameters***

$K=10^{9}\left[cell\right]$ , Tumor cell carrying capacity,

$c=0.00147\left[LCell^{1}Day^{-1}pmol^{-1}\right]$ , Tumor growth rate induced by E2,

$\alpha_{10}=0.2263\;\left[mg^{-1}\right]$ , Tumor growth inhibition by AZD9496,

$\alpha_{1}=0.507\;\left[Lpmol^{-1}\right]$ , Half saturation constant,

$\beta_{1}=7.08\cdot 10^{-8}\;\left[Cell^{-2}\right]$ , Half saturation constant,

$p_{1}=8.7\cdot 10^{-4}\;\left[L^{2}Cell^{-2}Day^{-1}\right]$ , NK induced tumor death,

$\alpha_{2}=7\cdot 10^{6}\;\left[Cell^{-1}\right]$ , Half saturation constant,

$\beta_{2}=5.4\cdot 10^{-5}\;\left[L^{2}Cell^{-2}\right]$ , Half saturation constant,

$\beta =6.3\cdot 10^{-3}\;\left[Day^{-1}\right]$ , WBC death rate,

$\alpha =5\cdot 10^{7}\;\left[CellL^{-1}Day^{-1}\right]$ , WBC production rate,

$e=0.00486\;\left[Day^{-1}\right]$ , Fraction of WBCs becoming NK cells,

$f=0.0693\;\left[Day^{-1}\right]$ , NK cell death rate,

$p_{2}=3.42\cdot 10^{-6}\;\left[CellDay^{-1}\right]$ , NK cell inactivation by tumor cells,

$p_{3}=1.87\cdot 10^{-8}\;\left[Cell^{-1}Day^{-1}\right]$ , NK cell recruitment rate

$\alpha_{3}=1.6\cdot 10^{-5}\;\left[Cell^{-1}\right]$ , Half saturation constant

$\beta_{3}=3.27\;\left[LCell^{-1}\right]$ , Half saturation constant

$p_{6}=2.04\cdot 10^{-3}\;\left[LCell^{-2}Day^{-1}\right]$ , CTL induced tumor death,

$\alpha_{6}=0.268\;\left[Cell^{2}\right]$ , Half saturation constant,

$\beta_{6}=4341\;\left[LCell^{-1}\right]$ , Half saturation constant,

$K_{L}=8\cdot 10^{8}\;\left[CellL^{-1}\right]$ , CTL carrying capacity,

$p_{5}=4.14\;\left[LCell^{-2}Day^{-1}\right]$ , CTL growth rate induced by IL-2,

$d0.41\;\left[Day^{-1}\right]$ , CTL death rate,

$\alpha_{5}=1000\;\left[Cell\right]$ , Half saturation constant,

$\alpha_{7}=24.3659\;Day^{-1}]$ , Absorption rate of AZD9496,

$\beta_{4}=4.7541\;\left[Day^{-1}\right]$ , Elimination rate of AZD9496,

$p_{4}=9\cdot 10^{-5}\;\left[Day^{-1}\right]$ , Fraction of naive CTL activated,

$\alpha_{4}=2.3\cdot 10^{-11}\;\left[gL^{-1}\right]$ , Half saturation constant,

$L_{N}=2.3\cdot 10^{8}\;\left[CellL^{-1}\right]$ , Naive CTL population,

$I=2.3\cdot 10^{-11}\;\left[gL^{-1}\right]$ , IL-2 concentration,

$\beta_{5}=0.64\;\left[Day^{-1}\right]$ , Elimination rate of palbociclib,

$\alpha_{8}=14.1512\;\left[Day^{-1}\right]$ , Absorption rate of palbociclib,

$\alpha_{9}=0.01\;\left[mg^{-1}\right]$ , Tumor growth inhibition by palbociclib,

$\alpha_{10}=0.2263\;\left[mg^{-1}\right]$ , Tumor growth inhibition by AZD9496,

$\epsilon_{i}=1.01$ dimensionless Free parameter.

## 3 Slow-fast subsystems, the singular perturbed vector fields method

The singular perturbed vector field method is presented in this section.

Generally, given a system of nonlinear differential equations, it is impossible to obtain an analytical solution in most cases. Various numerical methods can be applied to the system of nonlinear differential equations. However, the numerical solutions sometimes miss important and useless information especially when dealing with a mathematical model of cancer research. In addition, by applying numerical methods, graphs representing the solution can be generated, from which it is difficult to understand and draw conclusions from the solution profiles of the system and data on the system. Therefore, in most cases, applying asymptomatic methods or reduction methods that reduce the number of equations and investigating the “small” subsystems without losing important information about the entire system are preferred. However, to reduce the original system, the fast and slow variables of the system need to be determined; i.e., the exact hierarchy of the system of differential equations needs to be known and this is why standard asymptotic methods cannot be applied. Because in order to apply these methods, the mathematical model should be of the form of SPS system, i.e., the hierarchy of the system of equations should be exposed. Therefore, the primary objective of this section was to determine the hierarchy of the system. A transformation of the system to a new coordinate system will be applied. In the new coordinates, the hierarchy of the system will be revealed by calculating the eigenvalues and eigenvectors of the new system. This is the main aim of this section.

### 3.1 SPVFM

This section provides a detailed description of the SPVF method. The mathematical model has the dimension *n* = 10. The following steps are implemented

1: Select N vectors, $\text{Γ}=\left\{{\vec{x}}_{1},\ldots ,{\vec{x}}_{N}\right\}$ , ${\vec{x}}_{i}\in R^{n}$ , where N≫n .

2: Compute the mean value of the vector filed over the point from step 1 : $\bar{F}=\frac{1}{N}\sum_{i=1}^{N}\vec{F}\left({\vec{x}}_{i}\right)$ ,


$$\bar{F}=\left(\;\begin{array}{c} \frac{1}{N}\sum_{I=1}^{N}F_{1}\left({\vec{x}}_{i}\right)\\ \frac{1}{N}\sum_{I=1}^{N}F_{2}\left({\vec{x}}_{i}\right)\\ \vdots \\ \frac{1}{N}\sum_{I=1}^{N}F_{n}\left({\vec{x}}_{i}\right) \end{array}\;\right)=\frac{1}{N}\sum_{i=1}^{N}\left(\;\begin{array}{c} F_{1}\left({\vec{x}}_{i}\right)\\ F_{2}\left({\vec{x}}_{i}\right)\\ \vdots \\ F_{n}\left({\vec{x}}_{i}\right) \end{array}\;\right).$$

3: Define the following set: ${\text{Γ}}_{cs}=\{{\vec{x}}_{i}\in \text{Γ}:\left\|\bar{F}\left(x_{i}\right)\right\|>\left\|\bar{F}\right\|\}$ for simplicity let reindex ${\text{Γ}}_{cs}=\left\{{\vec{x}}_{1},\ldots ,{\vec{x}}_{N_{cs}}\right\}$ .

4: Build the ordered basis sets:

$B_{i}=\left\{{\vec{x}}_{\left(i-1\right)n+1},\ldots ,{\vec{x}}_{in}\right\}$ with the corresponding matrix


$$A_{i}=\left(\begin{array}{ccc} x_{1,\left(i-1\right)n+1} & \ldots & x_{1,in}\\ x_{2,\left(i-1\right)n+1} & \ldots & x_{2,in}\\ \vdots & \ldots & \vdots \\ x_{n,\left(i-1\right)n+1} & \ldots & x_{n,in} \end{array}\right)$$

and let $B=\left\{B_{1},B_{2},\ldots ,B_{m}\right\}$ , $A=\left\{A_{1},A_{2},\ldots ,A_{m}\right\}$ , where $m=\lfloor \frac{N_{cs}}{n}\rfloor$ .

5: Select only the reference basis set from step 4 which have $\left|Det\left(A_{i}\right)\right|$ above the average level over all determinate basis i.e., let $\text{Ω}=\frac{1}{m}\sum_{i=1}^{m}\left|Det\left(A_{i}\right)\right|$ , then the reference basis is $B_{rb}=\left\{B_{i}:\left|Det\left(A_{i}\right)\right|\geq \text{Ω},i=1,\ldots ,m\right\}$ . Again let us reindex, $B_{rb}=\left\{B_{1},B_{2},\ldots ,B_{k}\right\}$ with the matching reindex of vectors $\vec{x}$ .

6: For each i=1,2,…,k compute the eigenvalues of following matrix $T_{i}$ that correspond to the matching basis $B_{i}$ ,


$$T_{i}=\left(\begin{array}{ccc} F_{1}\left({\vec{x}}_{\left(i-1\right)n+1}\right) & \ldots & F_{1}\left({\vec{x}}_{in}\right)\\ F_{2}\left({\vec{x}}_{\left(i-1\right)n+1}\right) & \ldots & F_{2}\left({\vec{x}}_{in}\right)\\ \vdots & \ldots & \vdots \\ F_{n}\left({\vec{x}}_{\left(i-1\right)n+1}\right) & \ldots & F_{n}\left({\vec{x}}_{in}\right) \end{array}\right).$$

i.e., compute the determinant of the following matrix $\left|T_{i}-\lambda I\right|$ where I is the unit matrix, and solve the equation:


$$\left|T_{i}-\lambda I\right|=0$$

7: let $\left\{\lambda_{1}^{i},\lambda_{2}^{i},\ldots ,\lambda_{n}^{i}\right\}$ be in ascending ordered eigenvalues of $T_{i}$ . For each $T_{i}$ the maximum gap is computed as:


$$gap_{max_{i}}=\underset{n}{\max}\left(\left|\lambda_{n+1}^{i}\left(T_{i}\right)\right|/\left|\lambda_{n}^{i}(T_{i}\right|)\right).$$

8: Denote by $i_{max}$ the index for which $gap_{max_{i}}$ is maximal. Compute the eigenvectors of $T_{i_{max}}$ , i.e, solve the system of equations:


$$T_{i_{max}}-\vec{w}=0$$

and obtain the eigenvectors: $\left\{{\vec{w}}_{1}^{i_{max}},{\vec{w}}_{2}^{i_{max}},\ldots ,{\vec{w}}_{n}^{i_{max}}\right\}$ , that correspond to $\left\{\lambda_{1}^{i_{max}},\lambda_{2}^{i_{max}},\ldots ,\lambda_{n}^{i_{max}}\right\}$ consist of the desired coordinate system. Let $n_{s}$ - be the index for which $\left(\left|\lambda_{n+1}^{i_{max}}\left(T_{i_{max}}\right)\right|/\left|\lambda_{n}^{i_{max}}(T_{i_{max}}\right|)\right)$ is maximal. Then the vectors $\left\{{\vec{w}}_{1}^{i_{max}},{\vec{w}}_{2}^{i_{max}},\ldots ,{\vec{w}}_{n_{s}}^{i_{max}}\right\}$ and, $\left\{{\vec{w}}_{n_{s}+1}^{i_{max}},{\vec{w}}_{n_{s}+2}^{i_{max}},\ldots ,{\vec{w}}_{n}^{i_{max}}\right\}$ are the new slow and fast vectors of the slow and fast system correspondingly.

9: Rewrite the original system in the new coordinate using the eigenvectors $\left\{{\vec{w}}_{1}^{i_{max}},{\vec{w}}_{2}^{i_{max}},\ldots ,{\vec{w}}_{n}^{i_{max}}\right\}$ .

## 4 Analysis and results

In this section, the SPVF method was applied to a mathematical model for cancer treatment. The mathematical model was transferred to a new coordinate system, allowing the model to be split into fast and slow subsystems. These subsystems were studied, and equilibrium points were found and analyzed for stability.

### 4.1 Transformation the mathematical model to a new coordinates, eigenvalues, eigenvectors

In this section, the mathematical model is transformed to new coordinates using the eigenvectors of the vector field.

By applying the SPVF method to the system of Equations 1, 2, 3, 4, 5, 6, 7, 8, 9, 10, The following eigenvalues and eigenvectors were obtained:


(12) $$\begin{array}{l} \lambda_{1}=2645971.569,\lambda_{2}=7477.865,\lambda_{3}=4635.569,\lambda_{4}=7876.957,\\ \lambda_{5}=9666.424,\lambda_{6}=872.764,\lambda_{7}=563.466,\lambda_{8}=366.763,\\ \lambda_{9}=35.534,\lambda_{10}=2.077. \end{array}$$

According to the algorithm of the SPVF , the maximum gap is $gap_{max_{i}}=\frac{\lambda_{1}}{\lambda_{2}}=353.840$ . The corresponding eigenvectors are as follows:


(13) $$\begin{array}{l} {\vec{w}}_{1}={\left(5.667,\;1.536,\;0.419,\;2.644,3.728,\;1.012,\;4.137,\;4.123,\;2.466,\;3.532\right)}^{T}\\ {\vec{w}}_{2}={\left(2.667,\;2.588,\;1.688,\;3.997,\;3.476,\;4.366,\;4.477,\;5.266,\;4.373,\;5.464\right)}^{T}\\ {\vec{w}}_{3}={\left(0.488,-1.346,-2.037,-3.156,-2.134,-3.544,-0.348,\;4.478,\;4.377,\;0.743\right)}^{T}\\ {\vec{w}}_{4}={\left(2.334,-9.442,3.204,-1.378,\;4.326,\;5.466,-6.378,\;7.626,\;6.773,\;0.089\right)}^{T}\\ {\vec{w}}_{5}={\left(2.024,3.244,-2.387,\;2.377,-1.376,\;2.525,\;1.267,-3.987,\;2.337,\;0.377\right)}^{T}\\ {\vec{w}}_{6}={\left(0.876,\;0.870,\;0.875,\;0.346,-4.565,\;3.557,-3.765,-9.768,-2.975,-2.765\right)}^{T}\\ {\vec{w}}_{7}={\left(-1.121,\;0.032,\;0.578,\;1.897,\;2.543,\;7.523,-1.876,\;2.205,\;2.880,-8.255,\right)}^{T}\\ {\vec{w}}_{8}={\left(-0.786,-5.879,\;0.000,\;0.000,\;0.000,\;0.876,\;0.772,-0.012,\;0.772,\;0.766\right)}^{T}\\ {\vec{w}}_{9}={\left(0.877,-4.865,\;0.000,\;9.000,\;7.000,\;6.000,-1.099,-1.778,-9.765,-1.987\right)}^{T}\\ {\vec{w}}_{10}={\left(-0.000,4.765,\;3.865,\;3.544,-5.544,-5.346,-4.897,-0.463,-0.984,-1.987\right)}^{T}, \end{array}$$

where T denote the transpose operator. This means that the original system of equations can be decomposed into fast and slow subsystems, where the fast direction of the system is in the direction of the eigenvector ${\vec{w}}_{1}$ corresponding to the eigenvalue $\lambda_{1}$ , and the slow direction of the system is in the direction of the eigenvectors ${\vec{w}}_{2}-{\vec{w}}_{10}$ corresponding to the eigenvalue $\lambda_{2}-\lambda_{10}$ .

The next step of the SPVF method is to transform model (1-10) using the above eigenvectors; hence, let $\vec{W}$ be a vector of the dynamical variables of the mathematical model:

$\vec{W}=\left(C_{C},N_{K},W_{BC},C_{TL},A_{ZD}^{nc},A_{ZD}^{c},P_{a}^{nc},P_{a}^{c},ℱ,ℋ\right)$ and, $\vec{V}=\left(x_{1},x_{2},x_{3},x_{4},x_{5},x_{6},x_{7},x_{8},x_{9},x_{10}\right)$ be the variables of the model in the new coordinates. Hence, the system can be rewritten as


(14) $$\vec{V}=A\vec{W},$$

where the matrix A contains the eigenvectors obtained by applying the SPVF method.

The next step is to express the old system variables as functions of the new variables. To achieve this, multiply the set of Equations 14 by the inverse matrix of A


(15) $$\vec{W}=A^{-1}\vec{V}.$$

Take the derivative of the system (14) with respect to time:


(16) $$\frac{d\vec{V}}{dt}=A\frac{d\vec{W}}{dt}.$$

Then substitute the expressions of the RHS (right-hand side) from the system (1) $\hat{\text{a}}$ (10) instead of $\frac{d\vec{W}}{dt}$ in (16); that is,


(17) $$\frac{d\vec{V}}{dt}=A\frac{d\vec{W}}{dt}=A\vec{F}\left(\vec{W}\right),$$

Where


(18) $$\vec{F}\left(\vec{W}\right)={\left(F_{1}\left(\vec{W}\right),F_{2}\left(\vec{W}\right),F_{3}\left(\vec{W}\right),F_{4}\left(\vec{W}\right),F_{5}\left(\vec{W}\right),F_{6}\left(\vec{W}\right),F_{7}\left(\vec{W}\right),F_{8}\left(\vec{W}\right),F_{9}\left(\vec{W}\right),F_{10}\left(\vec{W}\right)\right)}^{T}$$

Finally, substitute Equation 15 into Equation 17 to obtain the original mathematical model in the new coordinates with the initial conditions as follows:


(19) $$\begin{array}{l} \frac{d\vec{V}}{dt}=A\cdot \vec{F}\left(A^{-1}\vec{V}\right)\equiv \vec{B}\left(\vec{V}\right),\\ \vec{V}\left(0\right)=A\vec{W}\left(0\right). \end{array}$$

The system of differential equations obtained (19) is a system of equations that only describes a mathematical model and has no biological or physical meaning because the new variables are a combination of the old variables without any expression meaning. However, the great advantage of this system is that in these new coordinates, the hierarchy of the system is precisely known; therefore, the fast and slow variables are exactly known. This procedure enables division of the new ODE system into fast and slow subsystems. The procedure for splitting into fast and slow subsystems is as follows:


(20) $$\left(\frac{dx_{1}}{dt},\frac{dx_{2}}{dt},\ldots ,\frac{dx_{10}}{dt}\right)=\left(\frac{1}{\epsilon }B_{fast}\left(\vec{x}\right),{\vec{B}}_{slow}\left(\vec{x}\right)\right),$$

Where $\vec{x}=\left(x_{1},\ldots ,x_{10}\right)$ is the vector of dynamical variables of the model in the new coordinates. As shown, variable $x_{1}$ is a fast variable of the new system that corresponds to the direction of the eigenvector corresponding to the largest eigenvalue *λ*_1_ obtained by applying the SPVF algorithm. This procedure allows us to reduce the system (19) written in new coordinates to only one significant subsystem (in this case, one equation). According to the theory of asymptotic analysis for a given system in the singular perturbed system (SPS) form, a fast subsystem can be studied while the slow system is frozen. When investigating a fast system, no important or relevant information regarding the entire system is lost. The main aim of this study was to determine the equilibrium points of the system and their stability. The advantage of the SPVF method is that the eigenvalues do not change in size under a linear transformation. Therefore, by determining the equilibrium points of the new system and analyzing their stability, an inverse transformation can be performed (using the inverse matrix of the eigenvectors) to find the equilibrium points of the original system and guarantee that these will be stable equilibrium points of the original mathematical model. The equilibrium point of the new system is determined by solving the following equation:


(21) $$\epsilon \frac{dx_{1}}{dt}=B_{fast}\left(\vec{x}\right),$$

for $\frac{dx_{1}}{dt}=0$ , i.e.,


(22) $$B_{fast}\left(\vec{x}\right)=0.$$

**Figure 1:**
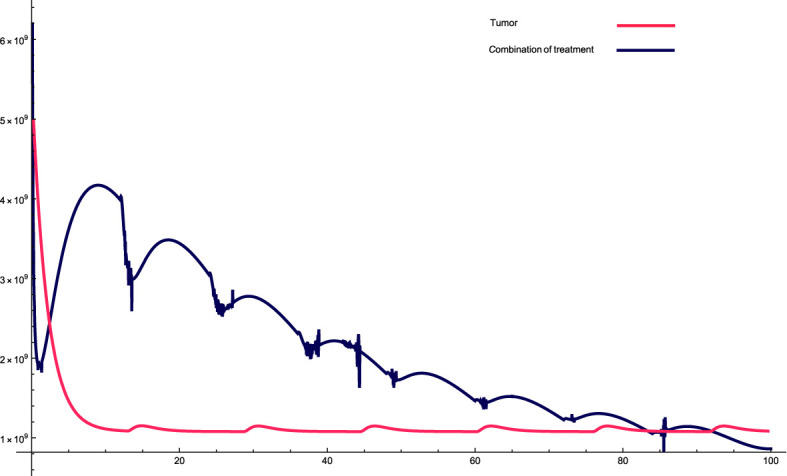
The solution profiles of the system of equations for a combination of parameters and the cancer cells stability. The parameters used for calculations are *K* = 10^9^ *, c* = 0.00147*, α*_10_ = 0.2263*, α*_1_ = 0.507*, β*_1_ = 7.08 · 10^−8^ *,α*_2_ = 7 · 10^6^ *, β*_2_ = 5.4 · 10^−5^ *, β* = 6.3 · 10^−3^ *,α* = 5 · 10^7^ *, e* =0.00486*,f* = 0.0693*, p*_2_ = 3.42 · 10^−6^ *, p*_3_ = 1.87 · 10^−8^ *, β*_3_ = 3.27 *p*_6_ = 2.04 · 10^−3^ *, α*_6_ = 0.268*, β*_6_ = 4341*, K_L_* = 8 · 10^8^ *, p*_5_ = 4.14*,d* = 0.41*, α*_5_ = 1000*, α*_7_ = 24.3659*, β*_4_ = 4.7541*, p*_4_ = 9 · 10^−5^ *, α*_4_ = 2.3 · 10^−^11*, L_N_* = 2.3 · 10^8^ *, I* = 2.3 · 10^−11^ *, β*_5_ = 0.64*, α*_8_ = 14.1512*, ϵ_i_* = 1.01.

**Figure 2:**
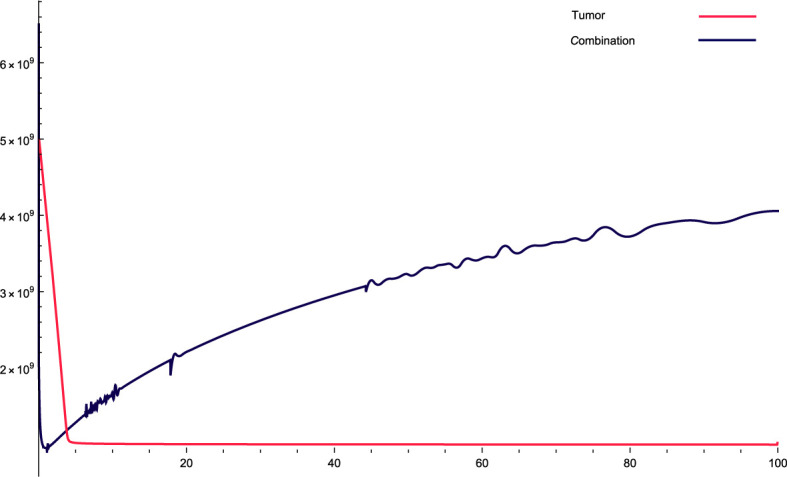
The solution profiles of the system of equations for a combination of parameters and the cancer cells stability. The parameters used for calculations are *K* = 7^6^ *, c* = 0.0633*, α*_10_ = 0.6467*, α*_1_ = 0.756*, β*_1_ = 2.89 · 10^−7^ *, α*_2_ = 6 · 10^4^ *, β*_2_ = 1.2 · 10^−5^ *, β* = 7.3 · 10^−3^ *, α* = 3 · 10^7^ *, e* = 0.646*, f* = 2.84739*, p*_2_ = 3.455 · 10^−5^ *, p*_3_ = 2.45 · 10^−8^ *, β*_3_ = 2.66 *p*_6_ = 2.76 · 10^−4^ *, α*_6_ = 3.766*, β*_6_ = 9875*, K_L_* = 5 · 10^5^ *, p*_5_ = 2.45*, d* = 0.65*, α*_5_ = 1240*, α*_7_ = 59.8735*, β*_4_ = 4.3*, p*_4_ = 9 · 10^−4^ *, α*_4_ = 5.5 · 10^−10^ *, L_N_* = 7.2 · 10^9^ *, I* = 1.9 · 10^−10^ *, β*_5_ = 1.89*, α*_8_ = 29.83*, ϵ_i_* = 0.1.

While the other variables of the system remain constant (frozen), they can be considered values of the initial conditions in the new system. As stated before, the mathematical model is transferred from the coordinates of the dynamical variables presented by the vector to new coordinates presented by the vector using the eigenvectors ${\vec{w}}_{i}$ . The transformation process involved expressing the new dynamic variables of the system as functions of the original model’s dynamical variables, i.e., the new variables are combinations of the old variables. The results are presented in **Figures 1** and **2** for different parameter values. In these figures, the black line represents the solution profile of the combination of the original variables, which cause the cancer cells to achieve stability. The red line represents the solution profile of cancer cells that achieve stability at the equilibrium point.

The parameters data that used in this research are presented in the relevant Figure. **Figure 1**: This combination of variables and parameters behaves in a roughly cyclic manner, meaning that it rises and falls; however, the general trend is downward. The intervals are approximately constant; however, the values on the y-axis vary. For this combination, the cancer cells stabilize at a relatively fast rate, meaning that there is a very sharp decrease at the beginning, which then reaches an equilibrium state very quickly. The sharp decrease at the beginning of treatment is attributed to the high combination of variables and parameters at the beginning of treatment, which causes a sharp decrease in cancer cells.

**Figure 3:**
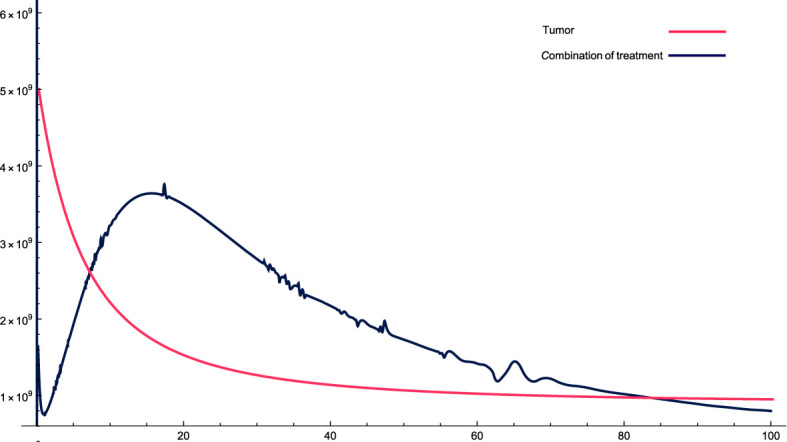
The solution profiles of the system of equations for a combination of parameters and the cancer cells stability. The parameters used for calculations are *K* = 7^6^ *, c* = 0.0633*, α*_10_ = 0.6467*, α*_1_ = 0.756*, β*_1_ = 2.89 · 10^−7^ *, α*_2_ = 6 · 10^4^ *, β*_2_ = 1.2 · 10^−5^ *, β* = 7.3 · 10^−3^ *,α* = 3 · 10^7^ *, e* = 0.00486*, f* = 0.766*, p*_2_ = 6.42 · 10^−5^ *, p*_3_ = 1.87 · 10^−8^ *, β*_3_ = 5.43 *p*_6_ = 2.76 · 10^−4^ *, α*_6_ = 0.688*, β*_6_ = 8765*, K_L_* = 5 · 10^6^ *, p*_5_ = 4.14*, d* = 0.41*, α*_5_ = 1400*, α*_7_ = 49.6533*, β*_4_ = 4.3522*, p*_4_ = 9 · 10^−5^ *, α*_4_ = 4.5 · 10^−11^ *, L_N_* = 1.2 · 10^8^ *, I* = 2.3 · 10^−11^ *, β*_5_ = 0.64*, α*_8_ = 14.6544*, ϵ_i_* = 0.09.

**Figure 3**: For these combinations of parameters and variables, it can be observed that, initially, cancer cells increase relatively sharply and then gradually fall, but not as in the previous case, where they fell cyclically. In this case, cancer cells do not decrease quickly and stabilize, but rather take relatively more time to stabilize.

**Figure 2**: For this combination of parameters, the values constantly increase in the dynamic variables of the original system. This indicates the aggressiveness of the treatment, that is, the variables increase over time with the treatment. With this combination, the cancer cells stabilized very quickly, indicating that they initially decreased very quickly and then stabilized in a straight step.

In all cases, a correlation is observed between the variables and parameters to the state where the equilibrium points stabilize.

### 4.2 Equilibrium points and stability analysis (46)

The equilibrium points of the mathematical model are examined in this section (written in the new coordinates) and their stability. In general, given a mathematical model presented by nonlinear ODE system, where the hierarchy of the variables is hidden, it is very hard and even impossible to study the stability of the equilibrium points analytically. Therefore, the great advantage of transforming the mathematical model to new coordinates is first of all exposing the hierarchy of the dynamic variables of the system, that is, of the mathematical model, subsequently, the model is split into a fast subsystem and a slow subsystem. by orders of magnitude of the eigenvalues ˆaaˆof the matrix that represents the vector field of the mathematical model. After splitting the mathematical model into subsystems, the fast subsystem can be analyzed, the equilibrium points can be found analytically in most cases as a function of the system parameters, while the other variables remain constant, and can be taken as the values of the initial conditions of the model. After finding the equilibrium points, their stability was analyzed, followed by an inverse transformation to the equilibrium points, based on principles from linear algebra, the equilibrium points and their stability are preserved under the linear transformation.

According to the results of the eigenvalues presented above the fast subsystem contain only the first variable i.e., $x_{1}$ while $x_{2},\ldots ,x_{10}$ are the slow variables.

The following steps are implements for finding the equilibrium points of the mathematical model in the new coordinates and determining their stability.

1. Substitute the slow variables as a constant into the fast subsystem (one can take the initial condition of the slow variables as the constants).

2. Setting the fast variable derivative to zero i.e., solve the fast subsystem (with slow variables as constants) and find the equilibria points of the fast variables $x_{1}^{*}$


$$B_{fast}^{1}\left(x_{1}^{*},x_{2}\left(0\right),x_{3}\left(0\right),\ldots ,x_{10}\left(0\right)\right)=0,$$

(where the star notation denoted the fast equilibrium points).

3. Substitute the equilibrium points from step 2( $x_{1}^{*}$ ) into the slow subsystem, solve the slow subsystem and find the equilibrium points of the slow variables $\left(x_{2}^{*},\ldots ,x_{10}^{*}\right)$


(23) $${\vec{B}}_{slow}^{2,\ldots ,10}\left(x_{1}^{*},x_{2}^{*},x_{3}^{*},\ldots ,x_{10}^{*}\right)=0$$

Here, the system consists of eight equations and eight unknown variables $\left(x_{3}^{*},\ldots ,x_{10}^{*}\right)$ .

**Figure 4:**
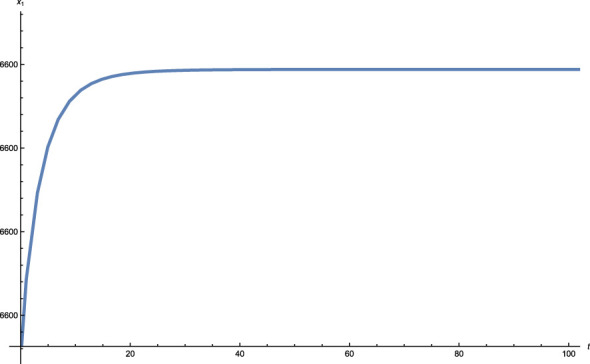
The solution profiles of the fast variable as a function of the time *t*.

4. Substitute the equilibrium points from steps 2 and 3 at the Jacobian matrix of the full system (the model at the new coordinates).

5. Compute the eigenvalues of the Jacobian matrix of the system (the model at the new coordinates) for each set of equilibrium point (the stable points are those with a negative real part of the eigenvalues).

6. Transform only the equilibrium points that are stable from steps 2 and 3 to the original coordinates using the inverse matrix of the eigenvectors, i.e., compute


(24) $${\vec{V}}_{stable}^{*}=A^{-1}{\vec{W}}_{stable}^{i*}$$

where *i* indicates for different stable equilibrium points. In **Figure 4** The solution profiles of the fast variable, depending on time *t*, are presented. As one can see from the plot graph the stability point reached after *t* ≈ 30 days. It is very important to note at this stage that the mathematical model in the new coordinates has no biological meaning since the new variables are a linear combination of the old variables. The only variables that have biological significance are the old variables. The important parameter from the stability analysis that can be extracted from the graph shown in **Figure 4** is time. An inverse linear transformation to the equilibrium points shows that after the same time parameter, both the cancer tumor and the whole system, as described by the mathematical model, stabilize.

## Conclusions

This study improves upon the mathematical model presented by (47), and added a system of differential equations that describe the treatment method depending on the dose and time of drug administration. The application of the SPVFM algorithm allowed for the reduction of the system’s dimensions. The model was then rewritten using the new coordinates to clearly expose its hierarchy. Representing the model in this manner allowed us to split the system into fast and slow subsystems. The fast subsystem was explored while the slow subsystem remained frozen in time. The equilibrium points of the fast subsystem were determined, and their stability was then studied. This information was transferred back to the original system of equations through an inverse transformation. A strong correlation was found between the combination of dynamic variables, system parameters, and cancer cells. The higher the values of the dynamic variables of the system, which also means that the more aggressive the treatment, the faster is the decrease in cancer cells, which tend to reach a state of equilibrium as quickly as possible.

## Data Availability

The original contributions presented in the study are included in the article/supplementary material. Further inquiries can be directed to the corresponding author.
